# XPS, structural and antimicrobial studies of novel functionalized halloysite nanotubes

**DOI:** 10.1038/s41598-022-25270-7

**Published:** 2022-12-14

**Authors:** Rashad Al-Gaashani, Yahya Zakaria, Ivan Gladich, Viktor Kochkodan, Jenny Lawler

**Affiliations:** Qatar Environment and Energy Research Institute (QEERI), Hamad Bin Khalifa University (HBKU), Qatar Foundation, 34110 Doha, Qatar

**Keywords:** Biochemistry, Biological techniques, Materials science, Nanoscience and technology

## Abstract

A novel robust preparation method based on thermal salt decomposition has been elaborated for synthesis of halloysite nanotubes (HNTs) impregnated with silver and iron oxide nanoparticles. The developed method is simple, time-effective, and can be employed for large scale material fabrication. Different characterization techniques, including X-ray diffraction (XRD), scanning and transmission electron spectroscopy (SEM and TEM) and energy dispersive X-ray spectroscopy (EDS) have been used to characterize the functionalized HNTs composite materials. Surface elemental and chemical state analysis was conducted using X-ray photoelectron spectrometer (XPS). The functionalized HNTs exhibit enhanced total surface area (by 17.5%) and pore volume (by 11%) compare to the raw HNTs calculated by using the Brunauer–Emmett–Teller (BET) method. It was shown that functionalized HNTs possess high antimicrobial properties towards both gram- positive and gram-negative bacteria species. The enhanced surface area and bactericidal properties of functionalized HNTs could be beneficial for employing of the prepared material as low cost filtration media for water treatment applications. Molecular dynamics (FPMD) were performed to obtain insights about possible physiochemical mechanisms for chemical adsorption and on the HNT thermal stability.

## Introduction

Development of nanomaterials with antimicrobial properties is an important field of research in material science, medicine, and environmental protection especially in water treatment^1–5^. Nowadays adsorption is one of the most attractive methods for water treatment. Many adsorbents are available in the market for instance activated carbon, zeolite, silica, kaolinite, montmorillonite, etc.^6–10^, however, there are still several issues related to lowsorption capacity and lack of antimicrobial properties of the commercial adsorbents. Therefore, development of novel low cost materials with improved performance and antimicrobial properties is of special importance for water treatment applications.

Over the last decades, there have been numerous attempts to enhance the sorption capacity and antimicrobial properties of the adsorbents^11–14^, however usually these works involve the employment of costly materials or very complex synthesis protocols that are difficult to adapt for large scale material production.

Halloysite (HNTs) is a cheap and widely accessible natural clay mineral. The chemical formula of HNTs is Al_2_(OH)_4_Si_2_O_5_·nH_2_O and it composed of multi-walled nanotubes built of tetrahedral (SiO) and octahedral (Al–OH) sheets^15^. It should be noted that the adsorption properties of HNTs are better than other clay minerals because of its large surface area, spiral-shape hollow tubular structure, basic and acid stability, higher cationic exchange capacity and higher reactivity of HNTs compared to other clays^16,17^. In addition, HNTs have a positive charge on the inner surfaces of the tubes that might facilitate the anions removal from water^18,19^.

Because of unique physicochemical properties such as large surface area, tubular nanostructure, mechanical strength, availability of functional groups and high biocompatibility HNTs is used in various applications such as a filler in polymeric materials, a carrier for drug delivery in medical and cosmetic formulations, tissue engineering and as absorbent for water treatment^19–22^. HNTs-based organic–inorganic composites properties and their applications in filters, microbe-resistant biocidal textile, medical formulations, drug delivery, paints and photocatalysis were previously studied and reviewed^23–26^.

Previously HNTs doping with silver has been studied^27–32^ as it is well known that silver doped materials posses strong antimicrobial activity against bacteria, viruses, and fungi^33–36^. Silver nanorods were synthesized inside the lumen of HNTs by thermal decomposition of the silver acetate by vacuum cycling^37^. Moreover, HNTs modification by iron oxide nanoparticles has been also reported in attempts to improve the adsorptive properties of the clay^19,38–43^, however, the HNTs based materials were prepared by using complex and time consuming approaches. As far as we know doping HNTs with Fe_2_O_3_ and Ag nanoparticles was not reported previously. Doping HNTs with Fe_2_O_3_ and Ag is aimed to have a functional synergy of both these nanoparticles. For instance, the anions removal by HNTs is not very effective due to electrostatic repulsion from the negatively charged outer surfaces of the tubes and HNTs doping with highly absorptive oxide surface as Fe_2_O_3_ can enhance the adsorption capacity of the HNTs^19^. HNTs doping with Ag will enhance the antimicrobial properties of the doped material.

In this work, we have used a novel thermal decomposition method for the synthesis of Fe_2_O_3_-Ag and HNTs-Fe_2_O_3_-Ag nanocomposite materials with antibacterial properties that can be used as filtration media in water treatment. It should be noted that the synthesis procedure is maximally adapted for scaling up of developed materials. To the best of our knowledge catalyst-free synthesis of HNTs + Fe_2_O_3_ + Ag nanocomposite is reported for the first time. Molecular dynamics simulations were accomplished to highlight possible physiochemical mechanisms for chemical adsorption of the different dopants and the stability of HNTs with temperature.

## Methods

### Materials

Iron (III) nitrate (Fe (NO_3_)_3_·9H_2_O, 99.99%), silver nitrate (AgNO_3_, ≥ 99.0%) and (HNTs) (H_4_Al_2_O_9_Si_2_.2H_2_O), LB agar, Miller were purchased from Sigma-Aldrich (St. Louis, Missouri, USA). Deionized water (DIW) of 18.2 MΩ/cm was used to prepare all aqueous solutions in experiments.

### Preparation of Fe_2_O_3_-Ag nanocomposites

Fe_2_O_3_-Ag nanocomposite materials were synthesized by a one-step thermal decomposition of Fe (NO_3_)_3_.9H_2_O and AgNO_3_ salts (2:1 wt%) in air using a muffle furnace (Thermo Scientific Thermolyne 5.8L A1 Benchtop Muffle Furnace, 240 V). In a typical preparation, 4 g of Fe(NO_3_)_3_·9H_2_O and 2 g of AgNO_3_ was mixed and dissolved in 20 ml of deionized water after 5 min stirring and 5 min sonication in a water bath. After that the solution was put inside a crucible and thermally treated at 450 °C for one hour. Then, the sample was cooling down to room temperature and collected as a pure powder that does not need to be washed.

### Preparation of HNTs doped with Fe_2_O_3_ and Ag

HNTs-Fe_2_O_3_-Ag composite materials were prepared by rapid thermal decomposition of Fe (NO_3_)_3_·9H_2_O and AgNO_3_ salts in the presence of HNTs under air conditions. In a typical preparation test, 1 g of Fe(NO_3_)_3_·9H_2_O and 0.5 g of AgNO_3_ were well mixed and dissolved in 7 ml of deionized water (5 min stirring and 5 min sonication in an ultrasonic bath). The mixture solution was then sprayed on 10 g of HNTs until fully absorbed by HNTs. The saturated HNTs sample was then treated at 450 °C for 60 min in the furnace and the cooled down to room temperature. Preparation steps to synthesize HNTs-Fe_2_O_3_–Ag composites are presented in the supporting information (Fig. S1).

### Characterization of the prepared composite materials

A Bruker D8 Advance X-ray diffractometer with Cu-Kα radiation source was used to obtain X-ray diffraction (XRD) patterns of the prepared materials from. Surface morphology of the samples was studied by using scanning electron spectroscopy (SEM) (JEOL JSM 7800F FE-SEM) and transmission electron microscopy of high resolution (HRTEM) (FEI Talos). The samples were put on carbon grids for TEM observations. Fast Fourier transform (FFT) images were optioned using a CCD camera of high resolution. The surface and porous properties of HNTs materials were determined by Brunauer–Emmett–Teller (BET) analysis with an ASAP-2020 surface analyzer. Degassing conditions were set at 200 °C for 480 min before BET measuring. Surface elemental and chemical state analysis was conducted using X-ray Photoelectron Spectrometer (XPS) Escalab 250Xi by Thermo Fisher Scientific UK. The spectrometer was calibrated using cleaned and high purity Au, Ag, Cu standards. The spectra were referenced using C main peak at 284.8 eV. The pass energy was 100 eV for survey scans and 20 eV for high resolution scans.

### Antimicrobial testing of HNTs-Fe_2_O_3_-Ag composite materials

The well-diffusion method was employed for evaluation of bactericidial properties of the prepared nanocomposites towards *Escherichia coli* (E. coli) and *Bacillus subtilis* (B. subtilis) species^44^. In this method the HNTs based materials were placed in Petri dishes, which contained the bacterial suspensions at the cells content of 120–200 cells per ml, and incubated at 37 °C for 24 h. If the prepared nanocomposites possess some bactericidal properties, the inhibition zones where the microbial growth is prevented are formed around the tested materials.

### Molecular Dynamics Simulation methodology

**Figure 1:**
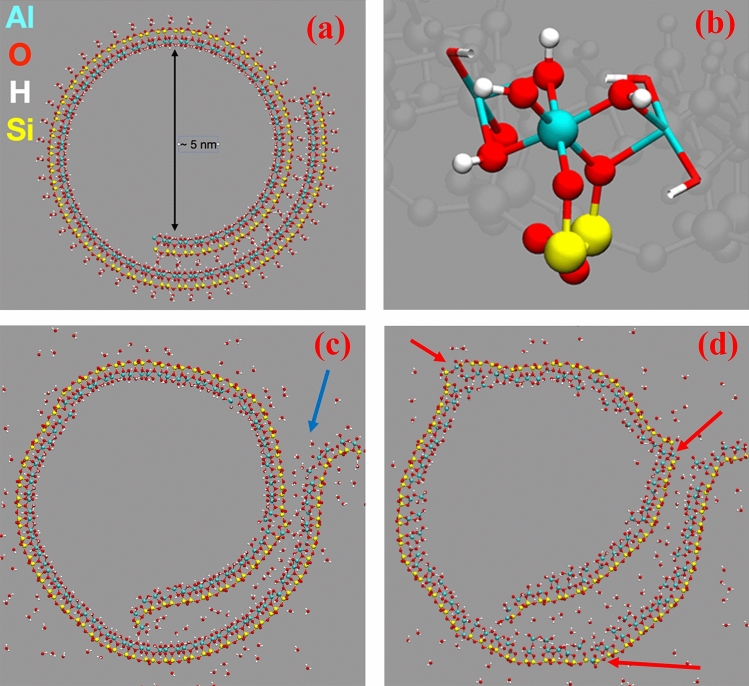
In (**a**), the optimized spiral HNT structure. In (**b**), a zoom on the structure showing the coordination of Al with 4 hydroxyl group and two –O–Si-. In (**c**) and (**d**), the structure at 300 and 800 K, respectively, after ~ 10 ps molecular dynamics. Atom color code Al (cyan), Si (yellow), O (red), and H (white). The blue arrow highlights the unfolding of the HNT spiral, the red ones the break of the HNT sheets.

An optimized structure of a spiral halloysite was downloaded from Ref.^45^ The structure comprised of a 1380 atom spiral and hydrated nanotube of a 10 Å A-type winding, with an inner cavity diameter of 50 Å (Fig. 1a). The spiral structure is hydrated by water molecules wetting the nanotube and also intercalated between the overlapping halloysite sheets. This spiral system has been centered in a simulation box of 300 Å by 300 Å by 5.2 Å dimensions in the X, Y, and Z, directions, respectively. Full periodic boundary conditions have been applied to all the three direction: the lateral X, Y dimensions provided sufficient empty spaces, resulting in the in modelling an infinite 2D spiral nanotube. Starting from the relaxed geometry, we performed two molecular dynamics simulations at constant volume and temperature at 300 K and 800 K. Simulations were performed using PM6 force matching, PM6-FM, semiempirical method^46^ as implemented in CP2K molecular dynamic package^47^. The time step was set to 0.5 fs and the convergence of the Self Consistent Field set to 10^–6^ Ha. Canonical sampling velocity rescale thermostat^48^ was adopted with a time constant of 300 fs. Similar methodology has been successfully used elsewhere^45^.

## Results and discussion

### Structure and morphology of HNTs materials

**Figure 2:**
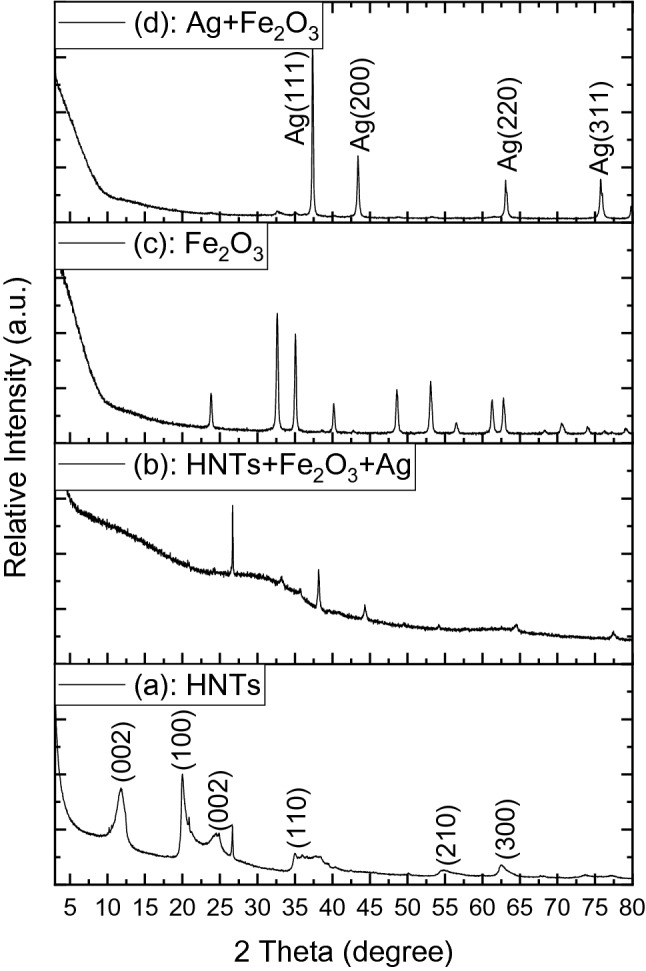
XRD spectra of raw HNTs (**a**), HNTs + Fe_2_O_3_ + Ag (**b**), Fe_2_O_3_ (**c**) and Fe_2_O_3_ + Ag nanocomposites (**d**).

Structural features of the raw HNTs and HNTs doped with silver and iron oxide samples have been studied by XRD as shown in Fig. 2. Figure S2a in the supporting information shows the XRD patterns of pure crystalline hematite phase (Fe_2_O_3_ with rhombohedral structure, JCPDS card No: 00-002-0915). The intense planes corresponding to hematite phase are (012), (104), (110), (113), (024), (116), (018), (214), (300), (208), (1010) and (217) located at 24.1, 33.3, 35.7, 40.9, 49.5, 54.2, 57.9, 62.7, 64.2, 69.3, 72.0 and 75.4 (2θº), respectively. However, the intense peaks corresponding to silver planes located at 38.1, 44.3, 64.5 and 77.4 (2θº) are (111), (200), (220) and (311), respectively (JCPDS card No: 01-087-0717) as illustrated in the supporting information Fig. S2b. It is clearly observed that the peak intensities of the Ag phase is significantly higher than the Fe_2_O_3_ phase^49^ as seen in Fig. S2b. Figure 2 presents XRD spectra of raw HNTs (a), HNTs doped with Fe_2_O_3_ and Ag (b), Fe_2_O_3_ (c) and Fe_2_O_3_–Ag nanocomposites (d). The raw HNTs has two phases: (1) a main phase (halloysite-7angstrom or aluminum silicate hydroxide with hexagonal structure, JCPDS card No: 00-029-1487) and (2) quartz (SiO_2_ with hexagonal crystal system, JCPDS card No: 00-005-0490). The intense peaks of raw HNTs are indexed with *Miller* indices (*hkℓ*) planes of hexagonal structure of aluminum silicate hydroxide as shown in Fig. 2a. The planes corresponding to quartz phase located at 20.8, 26.6, 36.5, 39.4, 50.16, 59.9, 68.1, 73.4 and 77.7 (2θº) are (100), (101), (110), (102), (112), (211), (203), (104) and (220), respectively (Fig. 2a). Figure 2b displays XRD data of four phases Ag, Fe_2_O_3_, SiO_2_ and very low halloysite phase. More information about the XRD pattern of raw HNTs can be found in the supporting information (Fig. S3).

**Figure 3:**
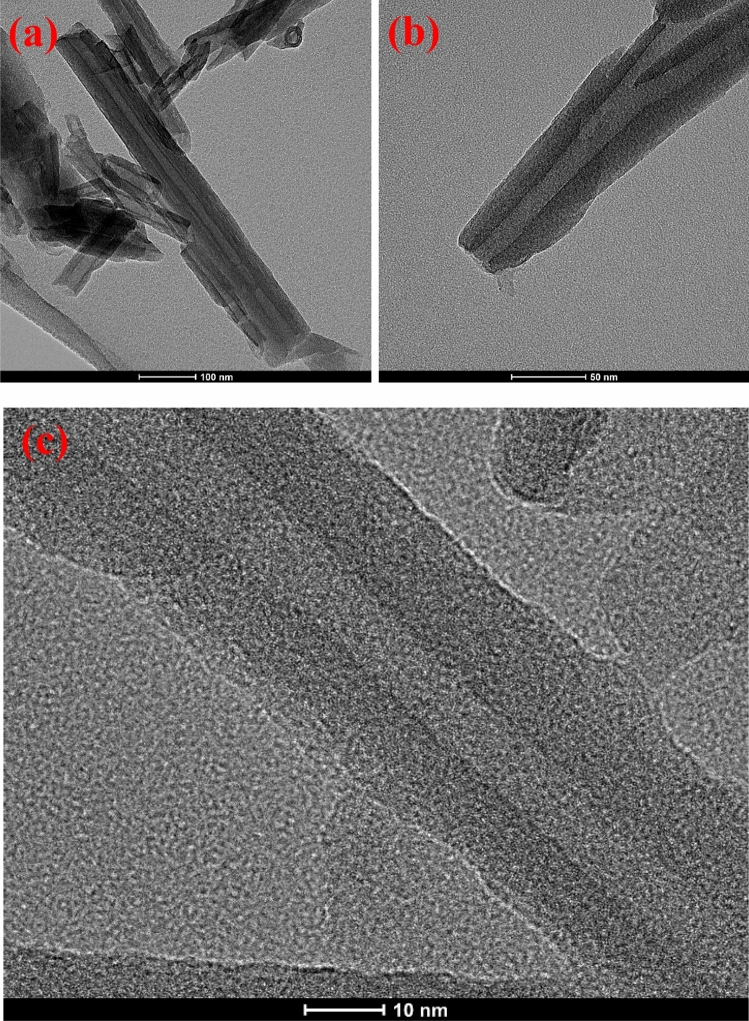
TEM images of raw HNTs before functionalization.

**Figure 4:**
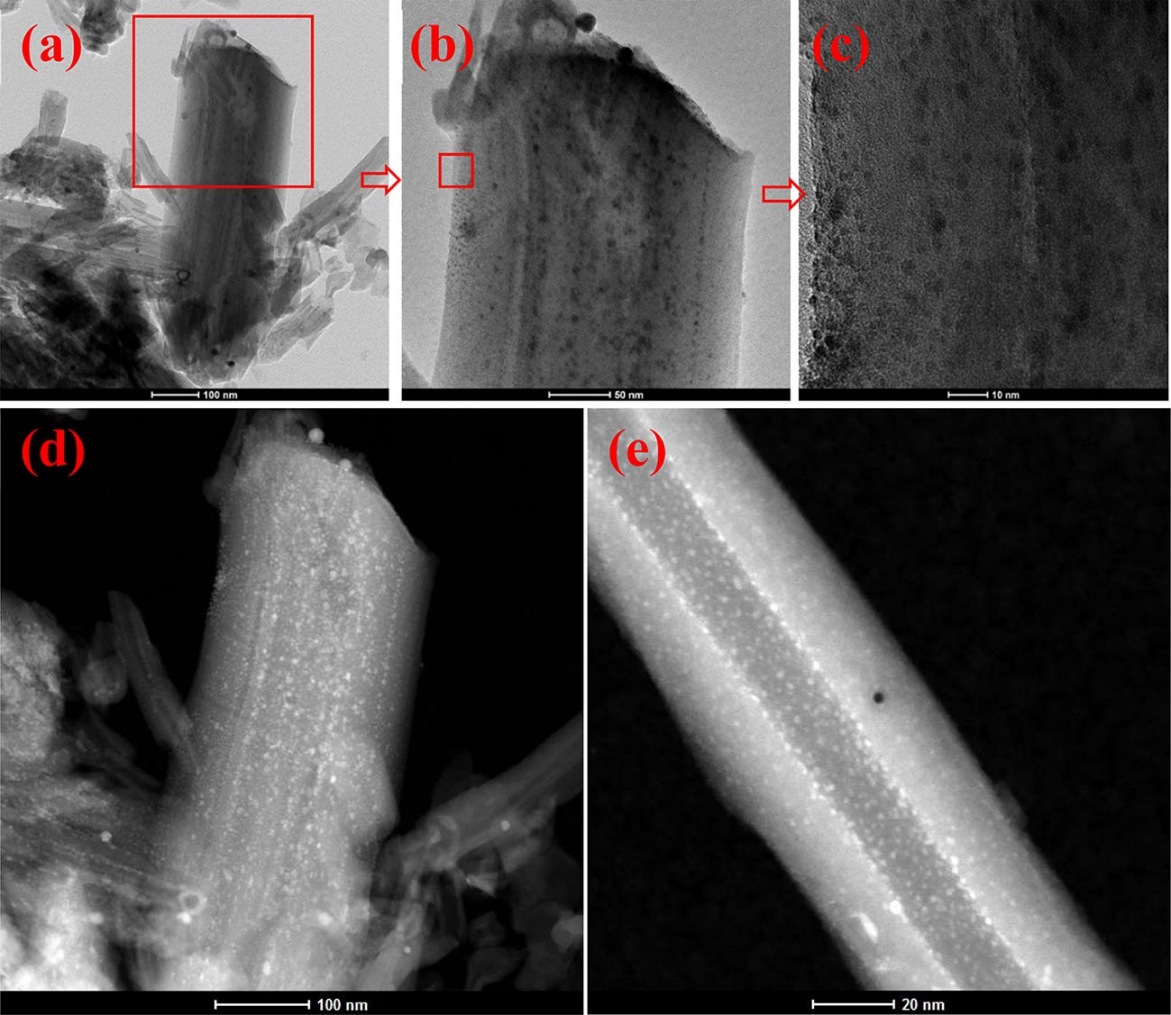
HR-TEM images of HNTs functionalized by iron oxide and silver nanoparticles.

The samples morphology was tested with SEM and TEM techniques. Fig. S4 in the supporting information shows SEM (a, b) and TEM (c–f) images of iron oxide doped with silver nanocomposite (2:1 wt%). The EDS mapping and EDS spectrum of Fe_2_O_3_-Ag nanocomposite show three main chemical elements of Fe, Ag and O (Fig. S5, in the supporting information). From SEM and TEM images (a–d), it is seen that the Fe_2_O_3_–Ag nanocomposite has a morphology like rugby ball with about 700 nm length and 350 nm width on average. This rugby ball shape results from the amalgamation of small iron oxide and silver nanoparticles. Fe_2_O_3_–Ag nanocomposites have quite developed surface and porous structure, which makes them suitable to be used as an absorbent medium for water treatment. Figure 3 shows TEM images of raw HNTs before functionalization. HNTs have open two-sided tubes that vary in length (Fig. 3a). The diameters of the outer tubes vary from 50 to 70 nm while inner diameters are about 10–15 nm and their wall thickness is about 18 to 22 nm on average (Fig. 3). It can be noted that the raw HNTs do not have any particles on its internal or external surfaces as shown in Fig. 3. However, there is a uniform distribution of iron oxide and silver nanoparticles on the functionalized HNTs after doping by iron oxide and silver as shown in TEM images (Fig. 4).

Some textural properties of raw and doped HNTs evaluated by BET analysis are displayed in Table 1. It is evident that, HNTs doping with iron oxide and silver notably increase the pore volume and surface area of the nanocomposite material, while decrease its pore size.

**Table 1:** Textural properties of raw and doped HNTs materials.

Textural properties	HNTs	HNTs + Fe_2_O_3_ + Ag
Total surface area (S_BET_) (m^2^/g)	57.0949	67.0544
Pore Volume: (cm^3^/g)	0.2565	0.2837
Pore Size: (Å)	157.953	146.067

**Figure 5:**
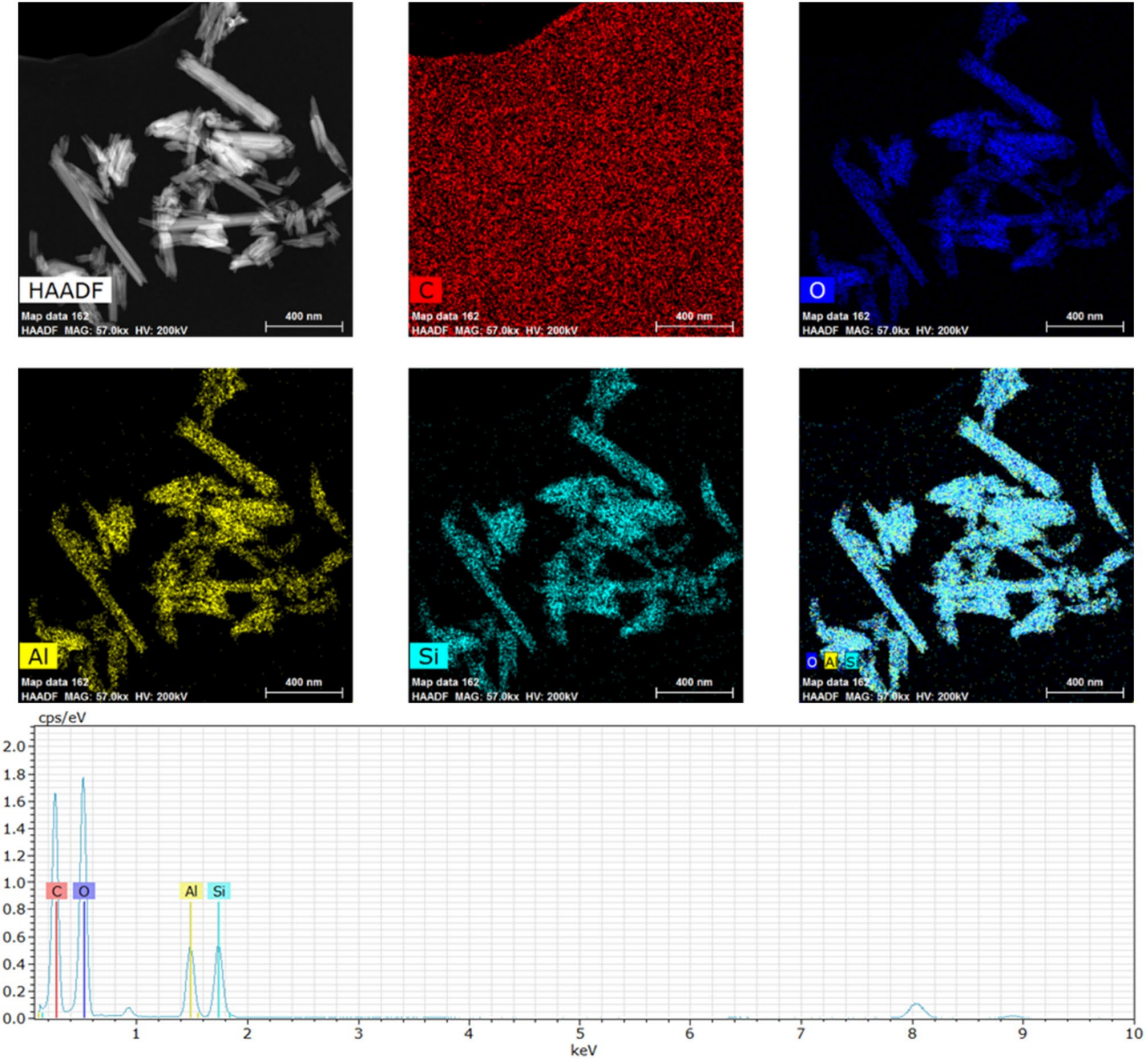
EDS mapping and EDS spectrum of HNTs before functionalization.

**Figure 6:**
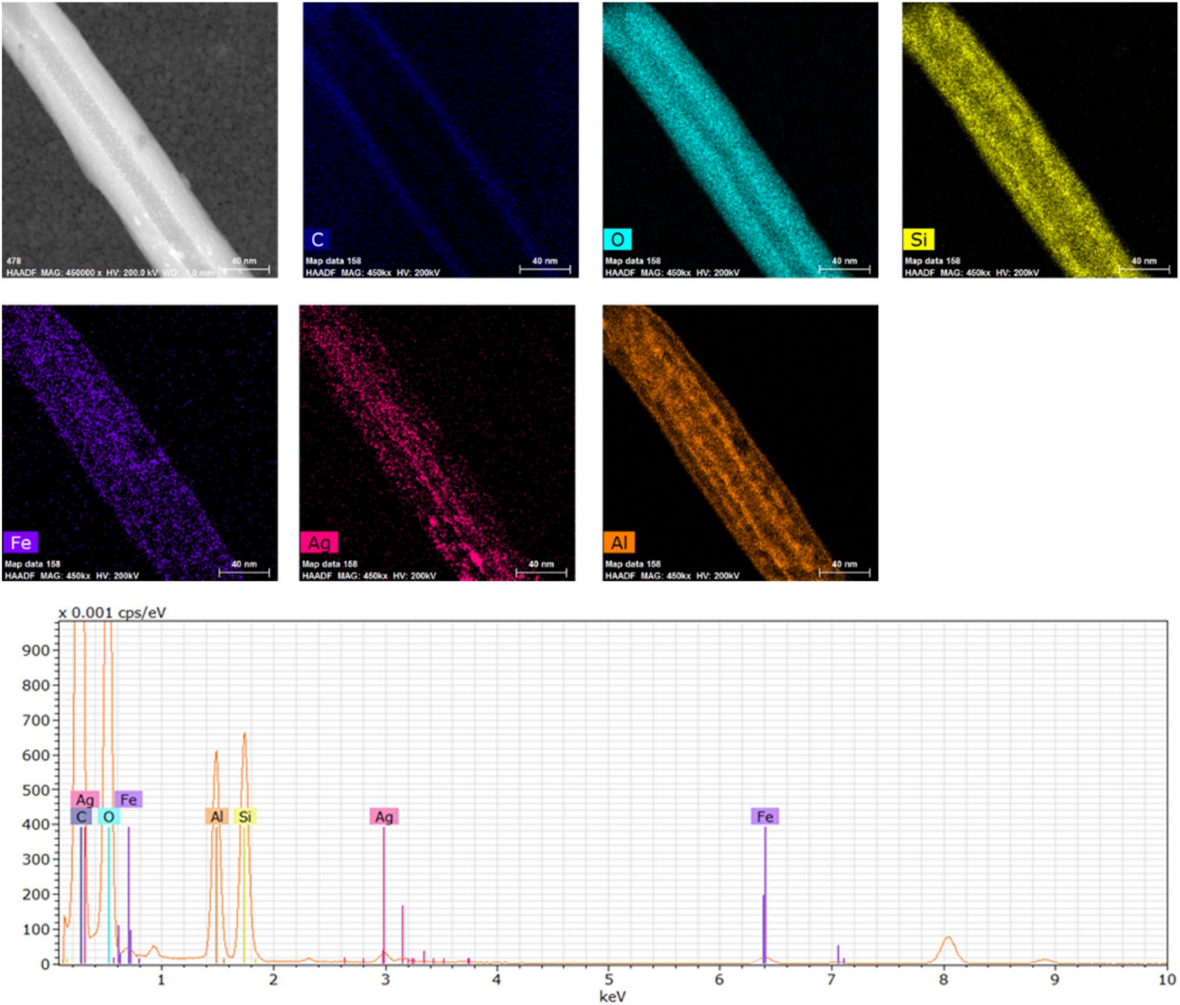
EDS mapping and EDS spectrum of HNTs functionalized by iron oxide and silver nanoparticles.

The EDS mapping and spectrum data confirm that the raw HNTs comprise mainly of Si, Al and O elements and the Si to Al wt% ratio is 1:1 (Fig. 5), while the main chemical constituencies of the HNTs functionalized by iron oxide and silver are Si, Al, Fe, Ag and O as shown in Fig. 6.

**Figure 7:**
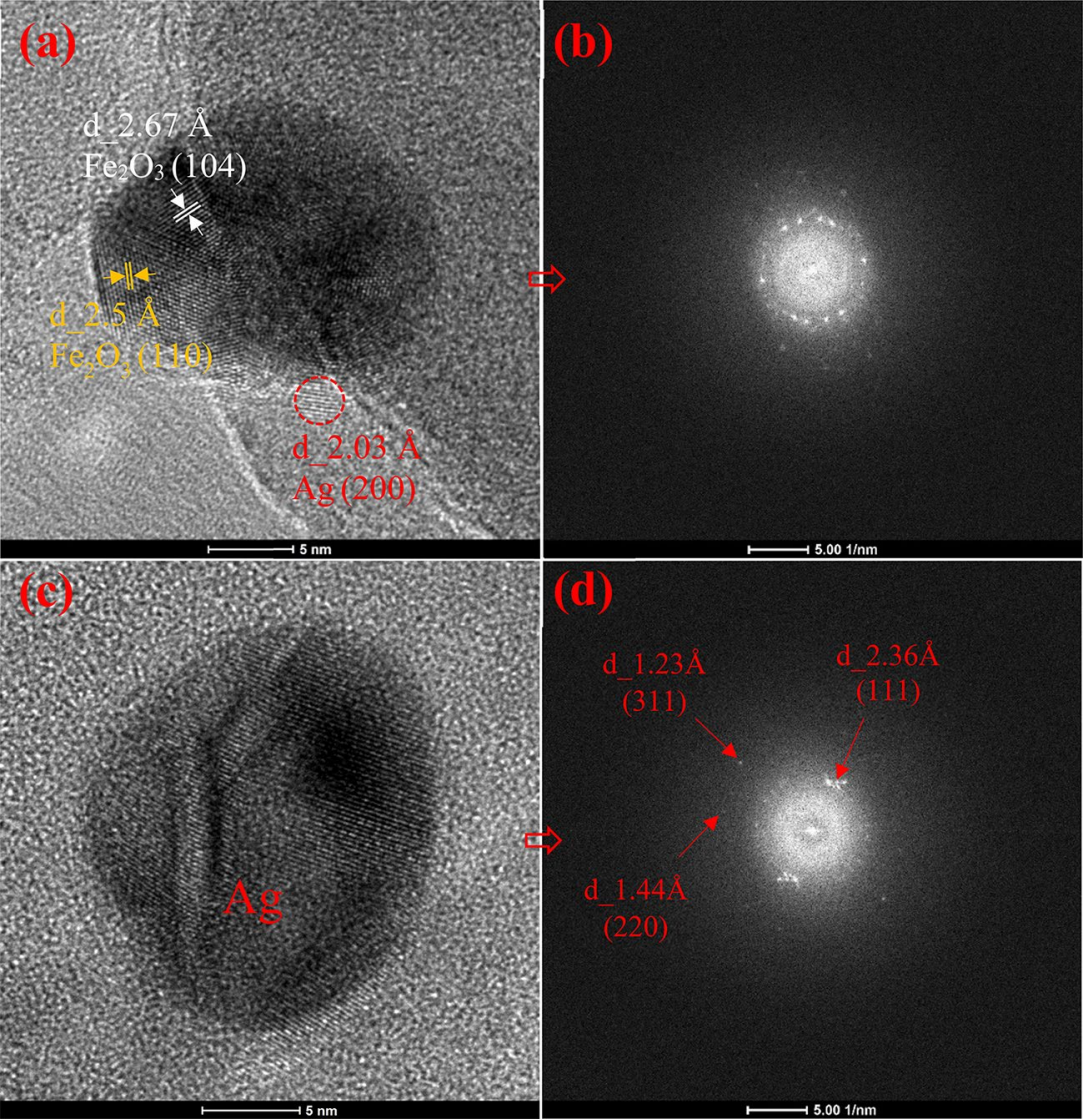
HRTEM images (**a** and **c**) and their corresponding FFT (**b** and **d**) of Fe_2_O_3_ and Ag nanoparticles, respectively. The corresponding d-spacing of Ag and Fe_2_O_3_ nanocrystals are shown.

Figure 4 shows the different sizes of HNTs which were regularly covered with silver and iron oxide nanoparticles. The sizes of silver and iron oxide nanoparticles are mostly less than 10 nm as shown in Figs. 4 and 7. HRTEM images of Fe_2_O_3_ and Ag nanoparticles and their corresponding FFT are shown in Fig. 7. FFT patterns show a lattice spacing of 2.5 Å corresponding to the (110) plane of the Fe_2_O_3_ crystal, which is in good agreement with the [110] growth direction. It can be seen from Fig. 7a that the Ag nanoparticle attached to the Fe_2_O_3_ nanoparticle (20 nm) has a size of less than 5 nm as shown in the circled zone. Figure 7a also shows d-spacing of 2.03 Å corresponding to (200) plane of Ag and 2.67 Å, 2.5 Å corresponding to (104), (110) planes of Fe_2_O_3_ crystal, respectively. The HRTEM results are in good agreement with XRD patterns.

### XPS study

**Figure 8:**
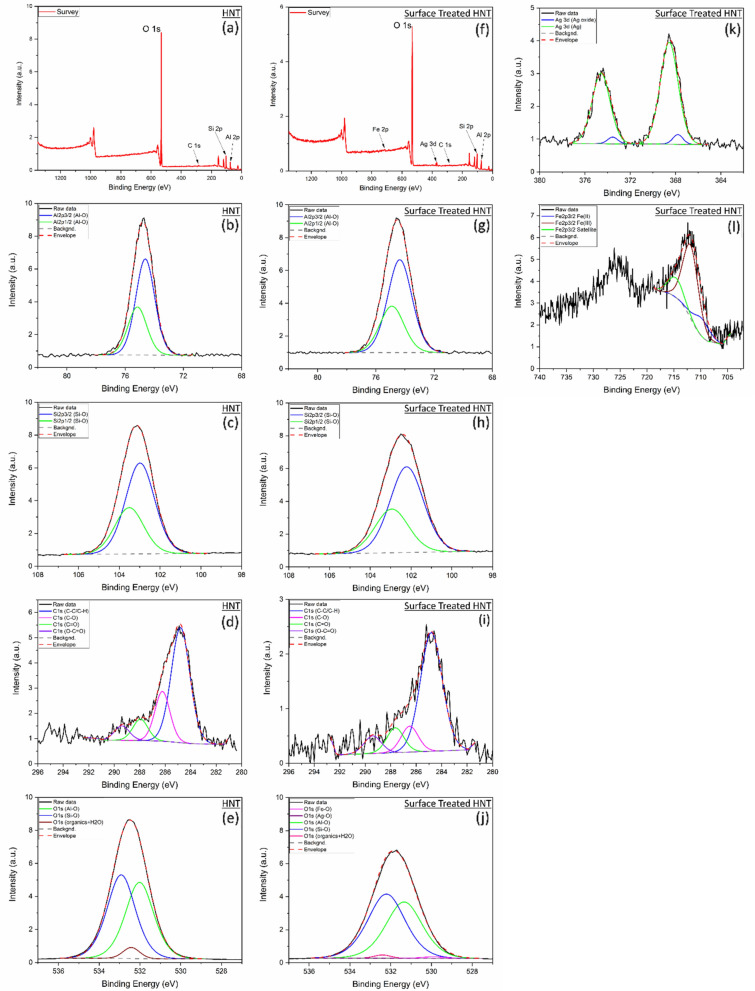
HNT XPS spectra including data fitting for (**a**) Survey, (**b**) Al 2p, (**c**) Si 2p, (**d**) C 1 s, (**e**) O 1 s. Surface treated HNT spectra including data fitting for (**f**) Survey, (**g**) Al 2p, (**h**) Si 2p, (**i**) C 1 s, (**j**) O 1 s, (**k**) Ag 3d, (**l**) Fe 2p.

The survey spectra quantification shows that the HNT sample has Al, Si and O as expected as well as C which is adventitious carbon (Fig. 8a). The atomic quantification is similar to the stoichiometry of H_4_Al_2_O_9_Si_2_. The O atomic concentration has slightly decreased after the HNT treatment and the ones for Si and Al has slightly increased which indicates a change of the stoichiometry at the surface namely oxygen reduction (Fig. 8f). The chemical state analysis and quantification for raw HNT and surface treated HNT are detailed in Table 2.

**Table 2:** Chemical state analysis and quantification for HNT and Surface Treated HNT.

Sample	Element	Survey	Chemical state analysis—spectra fitting				Total atomic (%)
		Atomic (%)	Peak BE (eV)	Area (%)	FWHM (eV)	Chemical state	
S1 (HNT)	Al (2p_3/2_)	13.9	74.6	–	1.5	Al–O	13.9
	Si (2p_3/2_)	16.3	103.0	–	1.7	Si–O	16.3
	C (1 s)	2.1	284.8	62.4	1.7	C–C/C–H (C=C)	1.3
			286.2	21.9	1.4	C–O	0.5
			287.9	9.4	1.4	C=O	0.2
			289.4	6.3	1.4	O–C=O	0.1
	O (1 s)	67.8	532.0	45.9	1.4	O–Al	31.1
			532.9	50.2	1.4	O–Si	34.0
			532.4	3.9	1	Organics/H_2_O	2.6
	Total	100.0	Total	100.0			
S2 (HNT + Fe + Ag)	Al (2p_3/2_)	14.9	74.4	–	2	Al-O	14.9
	Si (2p_3/2_)	18.4	102.2	–	1.9	Si–O	18.4
	C (1 s)	0.8	284.8	68.8	2	C–C/C–H (C=C)	0.5
			286.5	11.8	1.6	C–O	0.1
			287.6	11.3	1.6	C=O	0.1
			289.4	8.1	1.6	O–C=O	0.1
	Ag (3d_5/2_)	0.3	367.8	5.2	1.1	Ag–O	*0.0
			368.6	94.8	1.9	Ag	0.3
	O (1 s)	65.3	529.9	0.9	1.4	O–Fe	0.6
			530.5	0.0	1.4	O–Ag	*0.0
			531.3	45.3	2.1	O–Al	29.6
			532.2	52.6	2.2	O–Si	34.3
			532.4	1.2	1	Organics/H_2_O	0.8
	Fe (2p_3/2_)	0.4	709.8	8.4	3.4	Fe(II)-O	*0.0
			711.8	91.7	3.4	Fe(III)-O	0.4
	Total	100.0	Total	100.0			

*0.0 means the value is below 0.05%.

For HNT sample high resolution spectra fitting, Al 2p spectra revealed that Al 2p_3/2_ peak is positioned at 74.6 eV and Al 2p_1/2_ peak is positioned at 75.2 eV which indicates the presence of Al-O bonds. Si 2p spectra revealed that Si 2p_3/2_ peak is positioned at 103 eV and Si 2p_1/2_ peak is positioned at 103.5 eV which indicate the presence of Si–O bonds^50^.

For the high resolution spectra fitting related to Fe and Ag incorporated HNT, Al 2p spectra revealed that Al 2p_3/2_ peak is positioned at 74.4 eV and Al 2p_1/2_ peak is positioned at 74.9 eV which is slightly lower position than the pristine HNT. This lower binding energy along with the broadening of Al 2p peaks from 1.5 to 2 eV indicate that the increase of chemical disorder and defect formation in the HNT crystal structure which is mainly related to oxygen reduction and hydroxyl function deterioration. Si 2p spectra revealed that Si 2p_3/2_ peak is positioned at 102.2 eV and Si 2p_1/2_ peak is positioned at 102.9 eV which is lower position than the pristine HNTs. Similarly, this lower binding energy along with the slight broadening of Si 2p peaks from 1.7 to 1.9 eV indicate that the slight increase of chemical disorder and defect formation which is suggested to be related to oxygen reduction. The larger broadening of the peaks for Al 2p after HNTs treatment reveals that Al–O (mainly Al–OH) bonds at the internal layer of the HNT has been deteriorated compared to the outer bonds of Si–O. The outer layer formed by Si–O bonds in the halloysite nanotube has relatively kept it chemical structure. This is mainly due to stronger bonds of Si–O compared to Al–OH^51,52^. The shift to lower binding energy for Al 2p and Si 2p is likely due to the lower concentration of O, which was reduced after the thermal treatment, and eventually to the lower oxidation state.

Ag 3d spectra reveals that the main chemical state is metallic Ag and there is a minor chemical state related to the Ag oxide. Ag 3d_5/2_ chemical state analysis shows that the peaks at 368.6 eV and at 367.8 eV are related metallic Ag and oxide Ag, respectively^53,54^. Their estimated chemical state percentage are 95% for metallic Ag and 5% for oxide Ag.

Fe 2p spectra fitting was completed using a simplified model using a single peak for each chemical state due to the knowledge of the main chemical state acquired by other characterization techniques and discussed previously in this manuscript. It is worth noting that the more accurate model for transition metals 2p spectra is the calculated multiple fitting involving several peaks for a single chemical state^55^. As Fe 2p spectrum intensity for the treated HNT is relatively low, the detailed chemical state analysis was not performed for this work. The single peak fitting of Fe 2p_3/2_ is showing three peaks for Fe (II), Fe (III), and satellite peak and their peak positions are 709.8, 711.8 and 714.8 eV, respectively as shown in Fig. 8 and Table 2. The estimate percentage for Fe(II)-O is 8% and for Fe(III)-O is 92%^56,57^.

O 1 s spectrum analysis for HNT sample shows the presence of O–Al at 532 eV (45.9%), O–Si at 532.9 eV (50.2%), and other contamination such as organic compounds and moisture related peak at 532.4 eV (3.9%). The O 1 s spectrum analysis shows the presence of O–Al at 531.3 eV (45.3%), O–Si at 532.2 eV (52.6%), O–Fe at 529.9 eV (0.9%), O-Ag at 530.5 eV (*0.01%) and other contamination such as organic compounds and moisture related peak at 532.4 eV (1.2%). The binding energy has decreased after the thermal treatment for O–Al from 532 to 531.3 eV and O-Si from 532.9 to 532.2 eV, and the peaks related FWHM have increased for both peaks from 1.4 eV to above 2 eV as shown in Fig. 8 and Table 2. This is due to the lower oxidation state as discussed for Al 2p and Si 2p spectra analysis.

C presence in both samples has the typical adventitious carbon chemical distribution which is mainly C–C/C–H with minor presence of C–O, C=O and O–C=O^58,59^. The presence of C higher for the pristine HNT, however, this presence is less predominant in the HNT + Fe_2_O_3_ + Ag sample (Fig. 8).

### Atomic simulation study of HNTs

**Figure 9:**
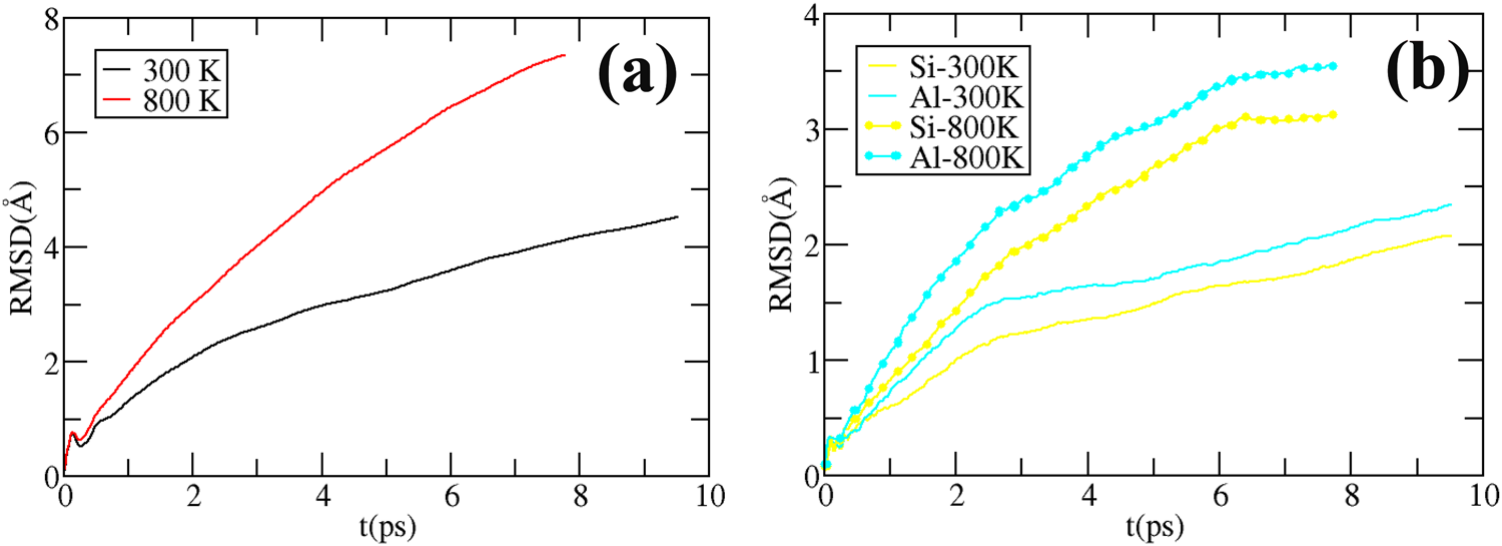
In (**a**) the root mean square displacement (RMSD) respect the starting structure, as a function of time for all the atom in the system and (**b**) for the Si and Al atoms only.

Figure 1 shows the initial and final structures at 300 and 800 K obtained after ~ 10 ps molecular dynamics. As observable in Fig. 1c,d, the folding of the HNT spiral is perturbed by the thermal motion of the intercalated water, which lead to a misfold of the spiral structure, especially at 800 K (Fig. 1d). Moreover, we report the break of the HNT sheet at 800 K, with the exposure of inner Al sites to the outer part of the HNT: this likely explains the higher chemical activity of the HNT at higher temperature. The root mean displacement (RMSD) from the initial starting structure provides a more quantitative description of the HNT behavior at different temperatures (Fig. 9). The RMSD suggests, as expected, that the structure undergoes to a larger rearrangement at higher temperature, Fig. 9a, but this structural arrangement is more important for the inner Al layer than for the outer Si one, as stated from the larger RMSD for Al than Si at the same temperature in Fig. 9b. To summarize, Figs. 1 and 9 suggest that the reactivity of the HNT is driven by an unfolding of the HNT spiral, and at higher temperature by the breaking of the HNT sheet and the exposure of Al sites.

### Antibacterial activity of HNTs based materials

**Figure 10:**
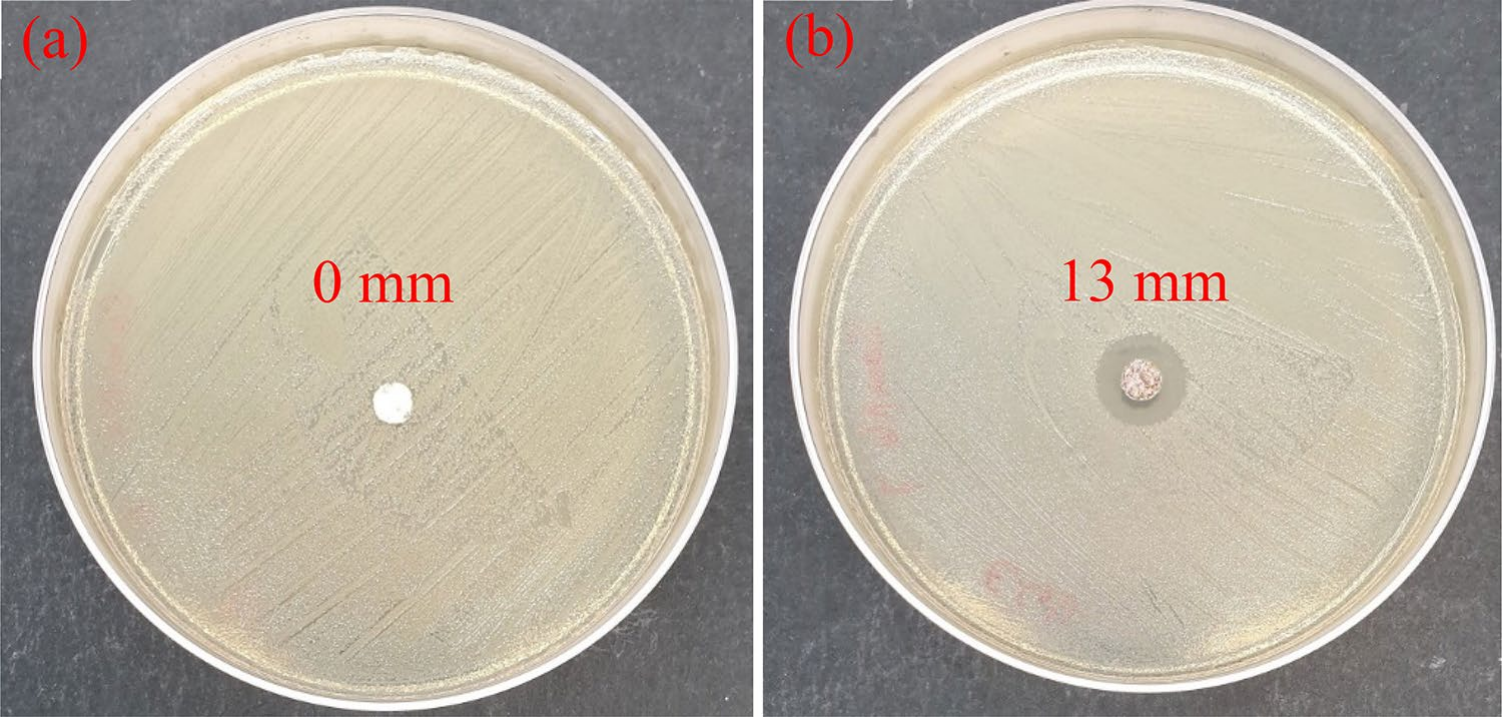
Bactericidal properties of (**a**) raw HNTs and (**b**) HNTs functionalized by iron oxide and silver nanoparticles towards the gram-negative *E.coli* bacteria.

**Figure 11:**
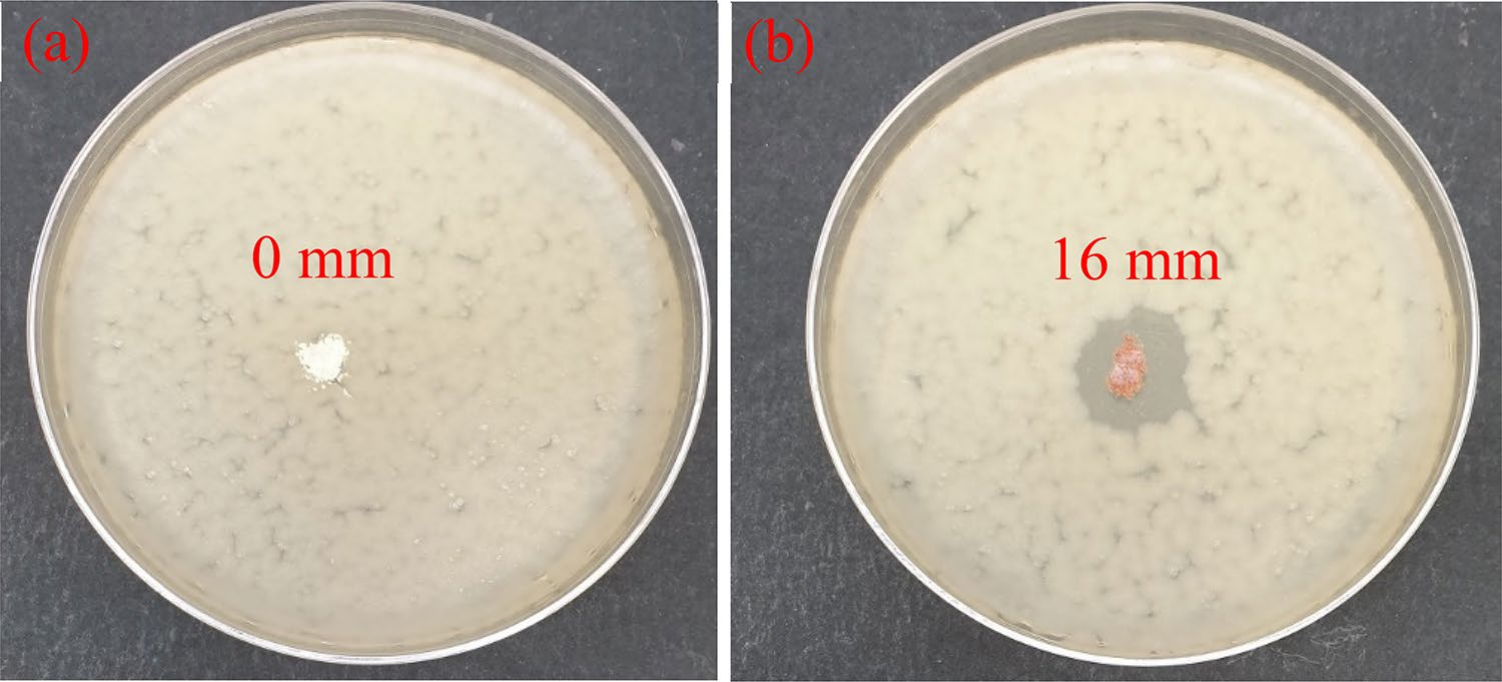
Bactericidal properties of (**a**) raw HNTs and (**b**) HNTs functionalized by iron oxide and silver nanoparticles towards the gram-positive *B. subtilis* bacteria.

Antibacterial properties of raw and doped HNTs materials towards *E*. *coli B. subtilis* bacteria have been studied by evaluation of the inhibition zone around the tested materials. As seen in Figs. 10a and 11a, the bacterial inhibition zones are lacking for raw HNTs. On the other hand, the inhibition zones for HNTs-Fe_2_O_3_–Ag composites against the used bacterial strains are visible in Figs. 10b and 11b: the diameter of inhibition zone for *E*.*coli* is 13 mm while 16 mm for *B*. *subtilis* bacteria.

These findings indicate that the prepared HNTs-Fe_2_O_3_–Ag materials are highly bactericidal towards the used microorganisms. The large diameters of inhibition zones can be explained by antimicrobial action of the doped silver. It should be noted that the silver-containing materials have found wide application as biocides because of their low toxicity to human^60^. Nevertheless, the precise bactericidal mechanism of silver incorporated materials is not fully clarified yet. It is suggested that silver nanoparticles adsorb, penetrate and damage the outer membrane of *E. coli* cell^61^. Rai *et al.*^62^ reported that silver ions released out of the materials matrix can inhibit the enzymes of the respiratory chain of the *E. coli* causing the cell death. It was proposed^63^ that the typical bactericidal action of silver- based materials includes (i) disorder of ATP synthesis in the bacterial cell by silver ions (ii) generation of reactive oxygen species by silver particles in the solution that damage the mitochondrial function of the cell lipids , and (iii) direct damage to the bacterial cell membranes by silver particles.

## Conclusions

In this study, for the first time a simple preparation method based on thermal salt decomposition was used for synthesis of HNTs impregnated by iron oxide and silver nanoparticles. The surface structure and morphology of the synthesized materials were investigated by using TEM and SEM methods. The successful doping of HNTs was confirmed by EDS chemical composition and XRD data. A uniform distribution of iron oxide and silver nanoparticles on HNTs surface was shown based on TEM and EDS data. It was found that HNTs doping with iron oxide and silver notably increase the total surface area (by 17.5%) and pore volume (by 11%) of the nanocomposites. Antimicrobial characteristics properties of the prepared functionalized HNTs were evaluated with both Gram-negative and Gram-positive bacteria such as *E. coli* and B*. subtilis*. Inhibition zones of large diameter of 13 and 16 mm were found for HNTs-Fe_2_O_3_-Ag composite towards *E. coli* and *B. subtilis* strains, respectively. These findings prove notable bactericidal effect of the prepared nanocomposite against the tested microbial cells. The strong antimicrobial properties, enhanced total surface area and pore volume of doped HNTs are promising for application of the prepared materials as filtration media in water treatment. It should be highlighted that the developed preparation method based on one stage thermal decomposition is simple, time efficient and well adapted for scaling up of developed materials.

## Supplementary Information

Supplementary Information.

## Data Availability

The datasets used and/or analysed during the current study available from the corresponding author on reasonable request.
