# Supplementary material for: XPS, structural and antimicrobial studies of novel functionalized halloysite nanotubes
Source: Sci Rep. 2022 Dec 14;12:21633. doi: 10.1038/s41598-022-25270-7 (PMC9751097; doi:10.1038/s41598-022-25270-7)
Supplement: Supplementary file 1 — Supplementary Information. [file 41598_2022_25270_MOESM1_ESM.docx]

**Supplementary material**

# **XPS, structural and antimicrobial studies of novel functionalized halloysite nanotubes**

Rashad Al-Gaashani^⁎^, Yahya Zakaria, Ivan Gladich, Viktor Kochkodan, Jenny Lawler

*Qatar Environment and Energy Research Institute (QEERI), Hamad Bin Khalifa University (HBKU), Qatar Foundation, 34110 Doha, Qatar*

****Corresponding author.*** *Tel: 0097430571456. E-mail:* [*ralgaashani@hbku.edu.qa*](about:blank)


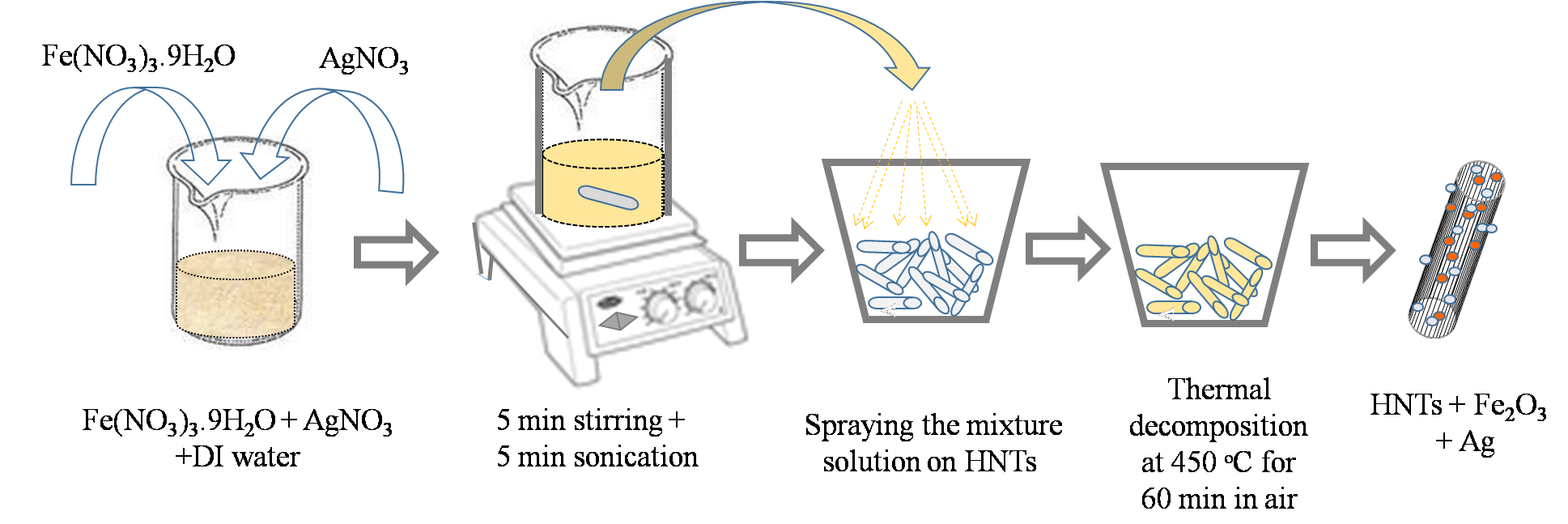


Figure S1. The schematic shows synthesis steps of HNTs-Fe_2_O_3_-Ag composites.


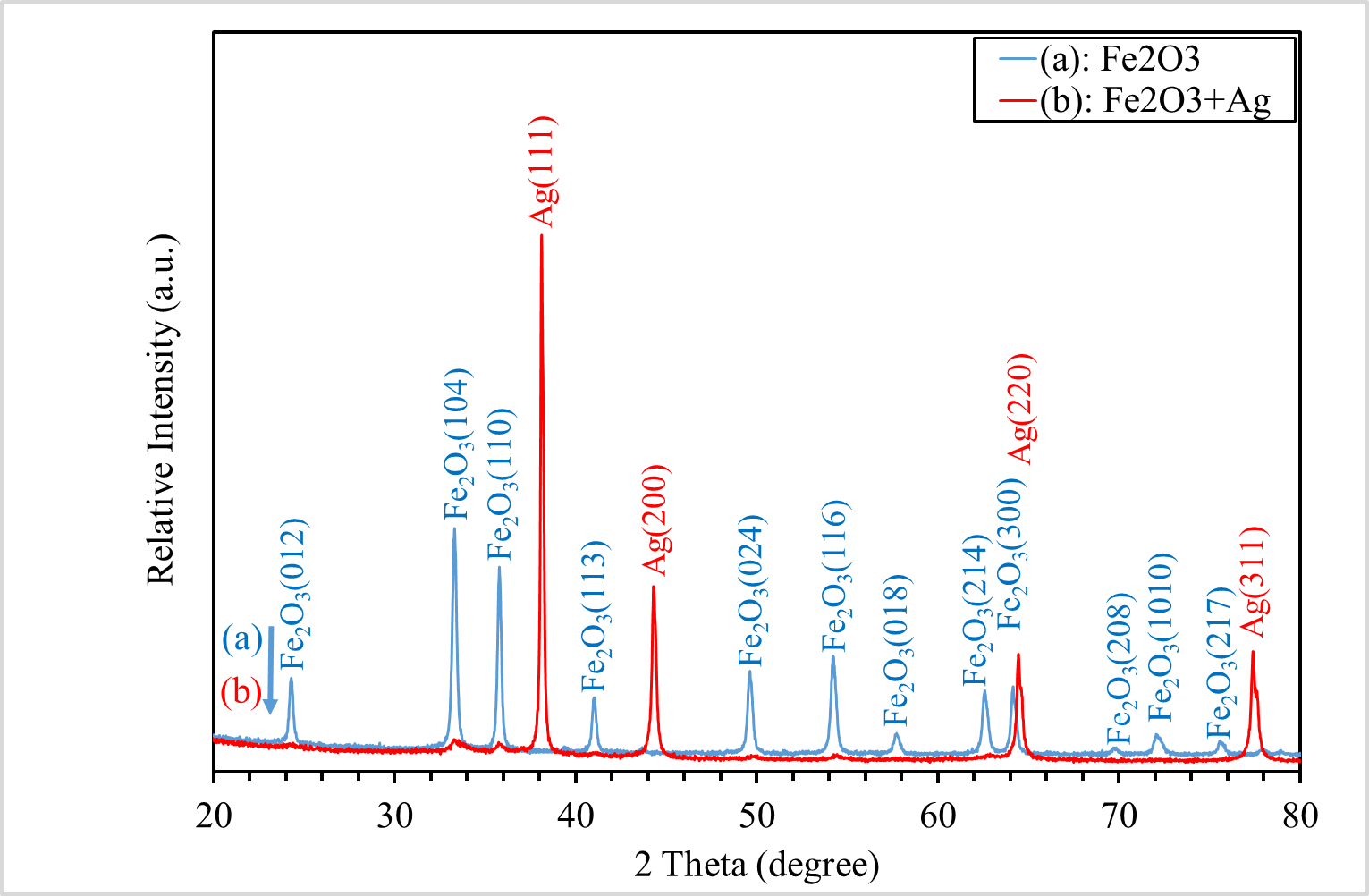


Figure S2. XRD spectra of Fe_2_O_3_ (a) and Fe_2_O_3_+Ag nanocomposites (b). It is clear that the intensity of iron oxide is much less than the silver intensity (b).

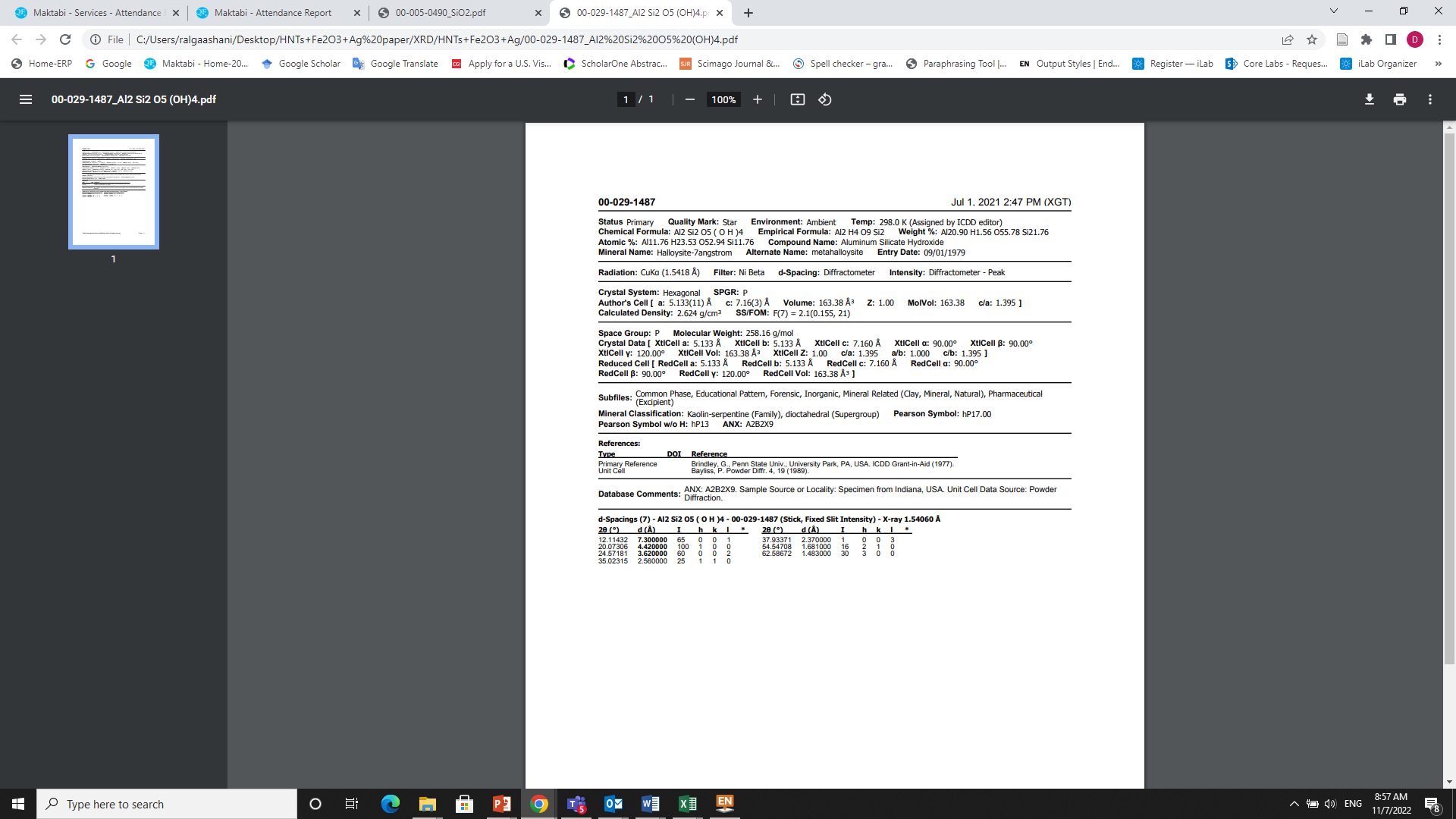


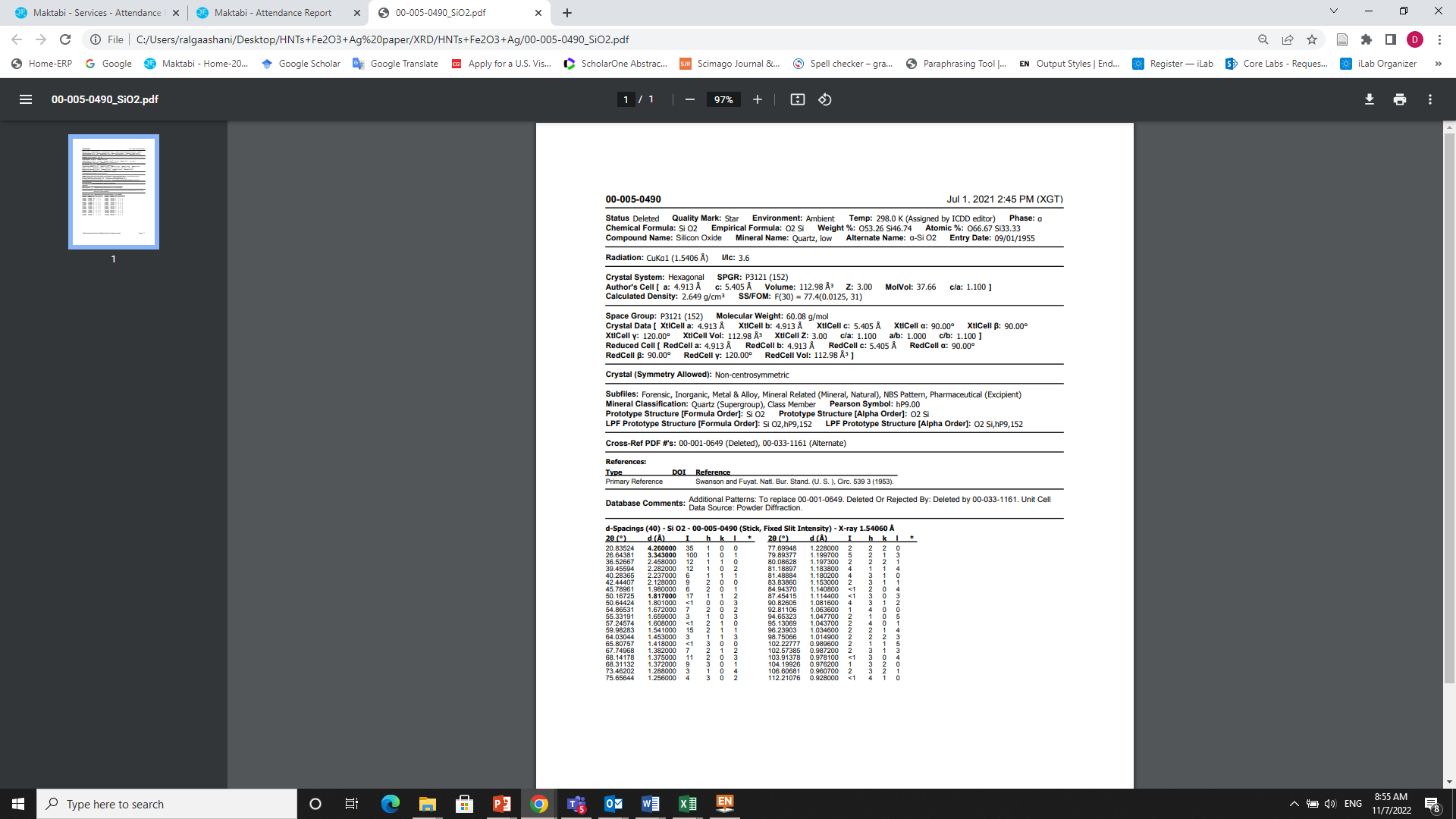


Figure S3. XRD pattern of raw HNTs showing two phases: halloysite or aluminum silicate hydroxide (PDF# 00-029-1487) and quartz (PDF# 00-005-0490).


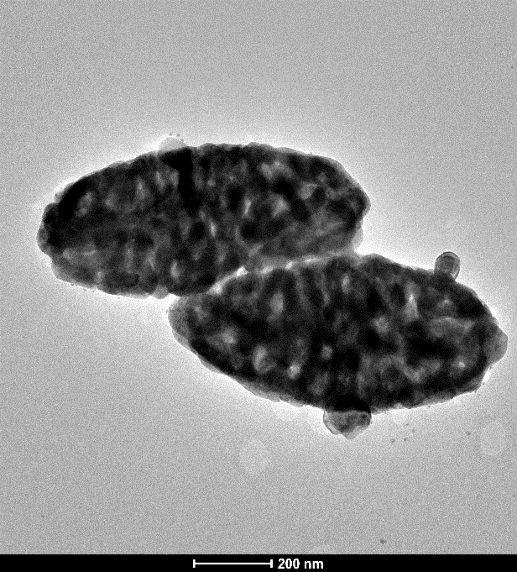

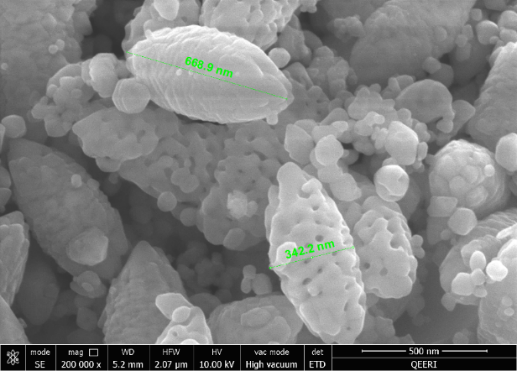

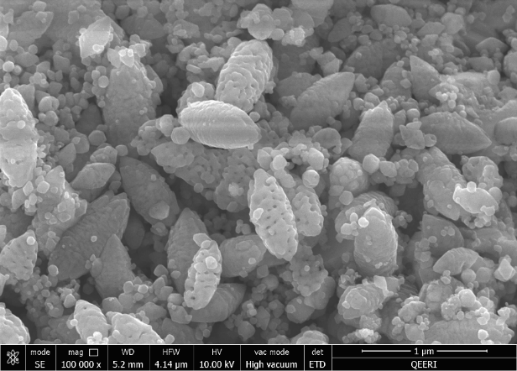

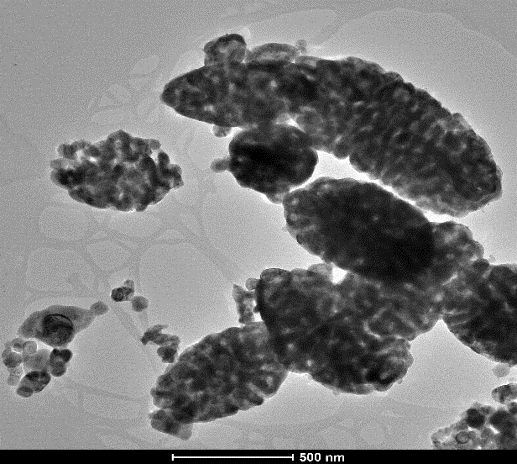

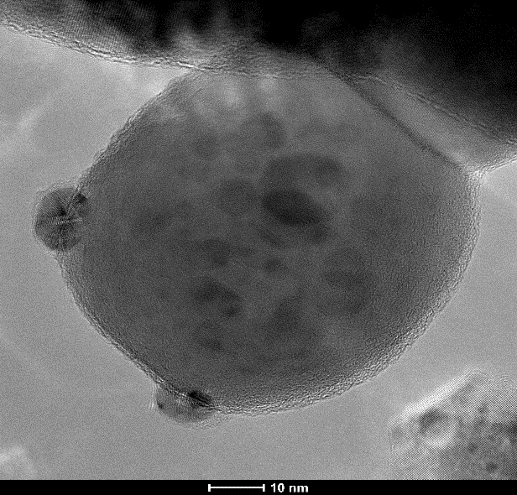

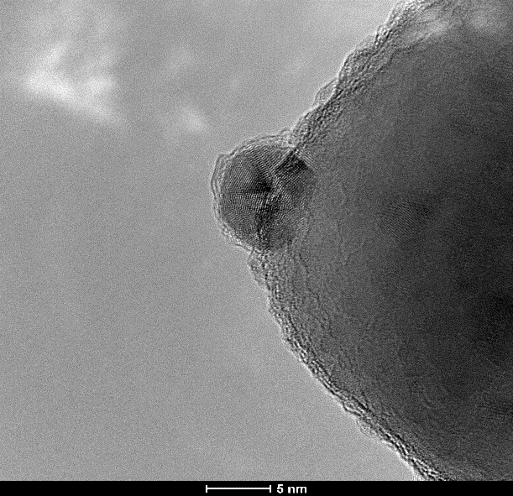


**(a)**

**(b)**

**(c)**

**(d)**

**(f)**

**(e)**

Figure S4. SEM (a, b) and TEM (c-f) images of Fe_2_O_3_-Ag nanocomposite


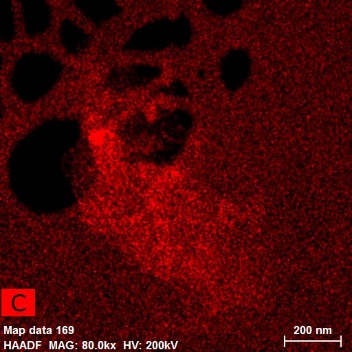

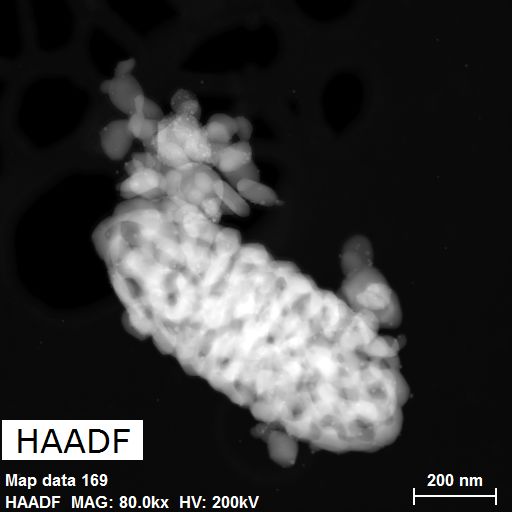

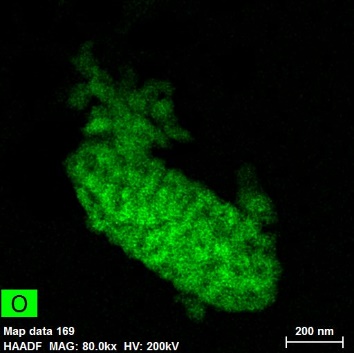

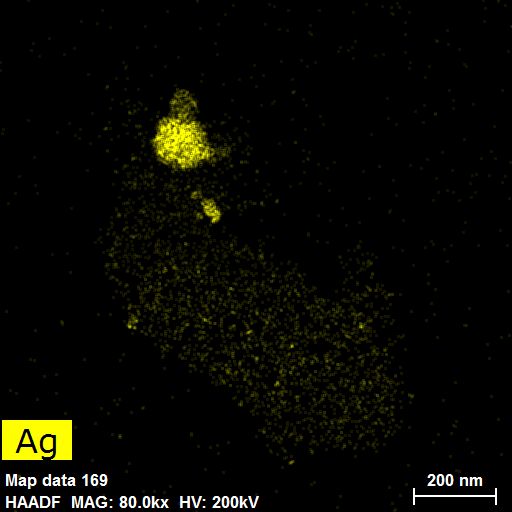

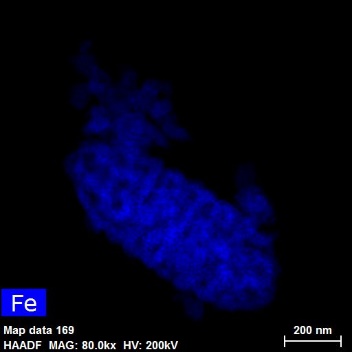

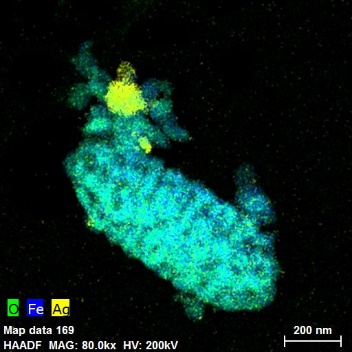

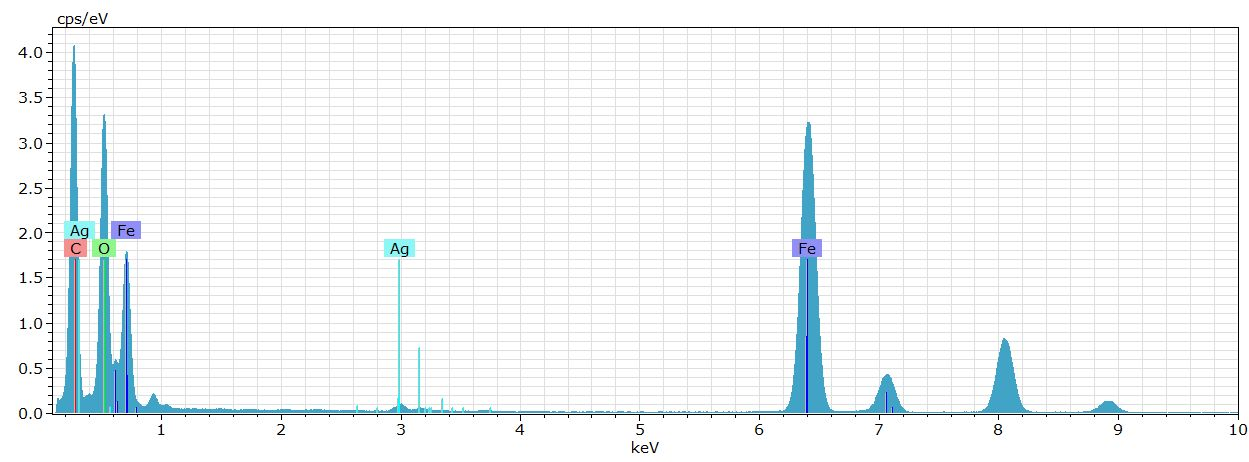


Figure S5. EDS mapping and EDS spectrum of Fe_2_O_3_-Ag nanocomposite.
